# Chronic, Mild Vestibulopathy Leads to Deficits in Spatial Tasks that Rely on Vestibular Input While Leaving Other Cognitive Functions and Brain Volumes Intact

**DOI:** 10.3390/life11121369

**Published:** 2021-12-09

**Authors:** Milos Dordevic, Sabrina Sulzer, Doreen Barche, Marianne Dieterich, Christoph Arens, Notger G. Müller

**Affiliations:** 1 Neuroprotection Lab, German Center for Neurodegenerative Diseases, Leipziger Str. 44, 39120 Magdeburg, Germany; sabrina.sulzer@st.ovgu.de (S.S.); notger.mueller@dzne.de (N.G.M.); 2 Department of Neurology, Otto von Guericke University, Leipziger Str. 44, 39120 Magdeburg, Germany; 3 Center for Behavioral Brain Sciences, 39106 Magdeburg, Germany; 4 Faculty of Health Sciences (FGW), University Potsdam, 14476 Potsdam, Germany; 5 Department of Otolaryngology, Head and Neck Surgery, Otto von Guericke University, 39120 Magdeburg, Germany; doreen.barche@med.ovgu.de (D.B.); christoph.arens@med.ovgu.de (C.A.); 6 Department of Neurology, Ludwig-Maximilians University, Marchioninistraße 15, 81377 Munich, Germany; marianne.dieterich@med.uni-muenchen.de; 7 Graduate School of Systemic Neuroscience (GSN), Ludwig-Maximilians University, 80336 Munich, Germany; 8 German Center for Vertigo and Balance Disorders DSGZ-IFB LMU, Ludwig-Maximilians University, 80336 Munich, Germany; 9 Munich Cluster for Systems Neurology (Synergy), 80336 Munich, Germany

**Keywords:** vestibulopathy, spatial, VBM, hippocampus, medial temporal lobe, balance, memory

## Abstract

Objectives: In this study, based on the known vestibulo-hippocampal connections, we asked whether mild chronic vestibulopathy leads only to vestibular-related deficits or whether there are effects on hippocampal function, structure, and cognition in general. In more detail, we assessed whether chronic vestibulopathy leads to (a) deficits in vestibular tasks without cognitive demand (balancing), (b) deficits in spatial cognitive tasks that require vestibular input (path integration, rotational memory), (c) deficits in spatial cognitive tasks that do not rely on vestibular input, (d) deficits in general cognitive function, and (e) atrophy in the brain. Methods: A total of 15 patients with chronic uni- or bilateral vestibulopathy (56.8 ± 10.1 years; 4 females) were included in this study and were age- and gender-matched by the control participants (57.6 ± 10.5) in a pairwise manner. Given their clinical symptoms and their deficits of the vestibulo-ocular reflex (VOR) the patients could be classified as being mildly affected. All participants of the underwent the following tests: clinical balance (CBT), triangle completion (TCT) for path integration, rotational memory (RM), the visuo-spatial subset of the Berlin intelligence structure test (BIS-4) and d2-R for attention and concentration, and a structural MRI for gray matter analysis using voxel-based morphometry (VBM). Results: Compared to the healthy controls, the vestibulopathy patients performed significantly worse in terms of CBT, TCT, and RM but showed no differences in terms of the BIS-4 and d2-R. There were also no significant volumetric gray matter differences between the two groups. Conclusions: This study provides evidence that both non-cognitive and cognitive functions that rely on vestibular input (balancing, path integration, rotational memory) are impaired, even in mild chronic vestibulopathy, while other cognitive functions, which rely on visual input (visuo-spatial memory, attention), are unimpaired in this condition, together with an overall intact brain structure. These findings may reflect a segregation between vestibular- and visual-dependent processes in the medial temporal lobe on the one hand and a structure–function dissociation on the other.

## 1. Introduction

Peripheral vestibular disorders are common in the older population, with rates of around 7% in people who are above 70 years of age [1]. Peripheral vestibulopathy is caused by an impaired or lost function of the vestibular hair cells in the labyrinth or of the eighth cranial nerve [2]. It is characterized by dizziness and imbalance while walking, which gets worse in dark environments, on uneven ground, or when the head is moving [3]. Other consequences that may deteriorate the quality of life of those who are affected include oscillopsia and the danger of falling [4].

Chronic vestibulopathy—a chronic vestibular syndrome that is characterized by unsteadiness when walking or standing due to vestibular hypofunction [3]—however, may lead to far more additional effects. The vestibular organs are connected to multiple sensory–motor brain areas as well as the medial temporal lobe memory system around the hippocampus [5,6,7] which receives vestibular input via multiple pathways [8]. In the hippocampus, both vestibular and visual input are integrated to form spatial memory representations, which are crucial for our ability to navigate in space. Vestibular damage entails a disruption in the spatial firing of the neurons that are located in the hippocampus [9,10] and a deficit in vestibular-based spatial memory and in the encoding of distance and direction [11,12,13] Vestibular function training can enhance performance in vestibular-dependent spatial memory tasks, whereas medial temporal lobe pathology leads to impairment in those same tasks [14,15,16] Regarding spatial memory that does not rely on vestibular but on visual input, findings in vestibulopathy patients are inconsistent (for a review see [17]). This is discrepancy might have been driven by the varying extent of damage in the vestibular system across studies, at least in part. 

Along with the reported functional deficits that are related to chronic vestibulopathy, some studies have found structural brain changes in affected patients as well, namely reduced hippocampal volumes relative to healthy controls [18,19]. Here, the extent of damage also influenced the results that were achieved, and patients with a (partial) bilateral vestibulopathy showed more pronounced effects than those with unilateral damage [20] and their lesions included the posterior parahippocampus [21] and sometimes even regions outside of the medial temporal lobe [22]. However, others failed to observe such differences [13,19] and instead reported a clear dissociation between structural and functional alterations in rats [23]. Regarding spatial memory that does not rely on vestibular but on visual input, findings in vestibulopathy patients are inconsistent, too (for review see [17]). Moreover, there are contrary results on spatial memory and hippocampus atrophy in unilateral deficits: while some authors could not find any changes [13], others demonstrated visuospatially impaired memory [24] and an atrophy of the posterior hippocampus in chronic deficits (after 2.5 years [25]).

To sum up, until now, it is unclear to what extent chronic vestibulopathy affects hippocampal function and structure. Hence, in an attempt to shed more light onto this issue, we set out to re-investigate the effects of chronic vestibulopathy on vestibulo-noncognitive, vestibulo-cognitive, visuo-cognitive, and general cognitive functions and brain volumes. In more detail, we hypothesized that patients with a history of proven uni- or bilateral damage to the peripheral vestibular system will show deficits in comparison to their pairwise-matched healthy controls on the following test batteries: (i) vestibular non-cognitive—widely used clinical balance test (CBT) [15,26] (ii) vestibular cognitive—triangle completion (TCT) and rotational memory (RM) [15,26], (iii) visual (non-vestibular) cognitive—visuo-spatial memory and visual construction (BIS-4) [27], (iv) general cognitive—concentration task (D2-R) [28,29], and (v) structural volumetric brain changes as assessed by voxel-based morphometry (VBM) [14]. With this extensive test battery, the functional effects of chronic peripheral vestibular damage could be assessed in a comprehensive and systematic manner.

## 2. Materials and Methods

### 2.1. Ethical Approval

This study was approved by the Ethics Committee of the Otto von Guericke University (approval number: 156/14). All subjects gave written informed consent in accordance with the Declaration of Helsinki.

### 2.2. Participants

**Figure 1:**
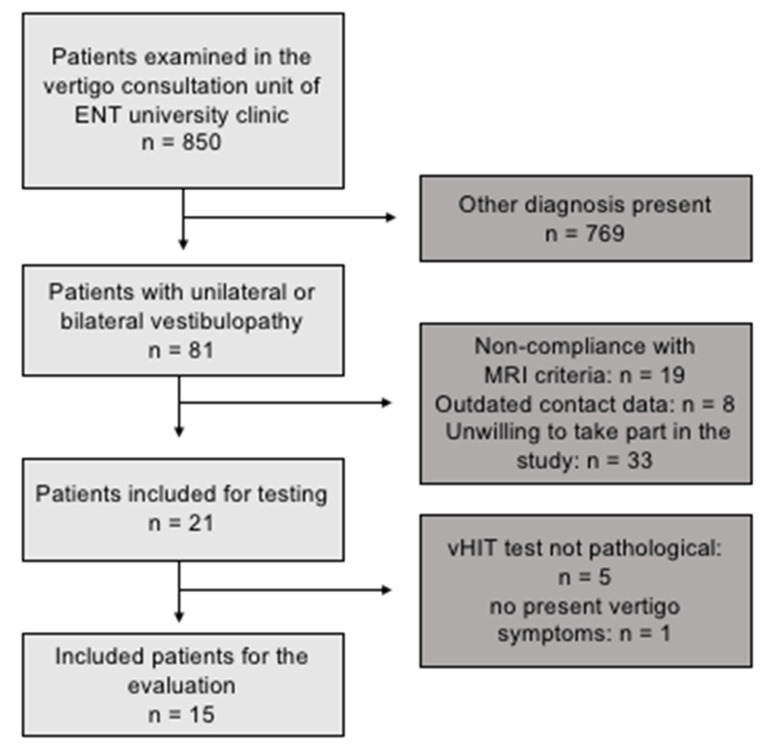
Patient recruitment flowchart.

The University Clinic of Otolaryngology, Head and Neck Surgery of Otto von Guericke University Magdeburg, provided the data from 850 patients (Figure 1) who had been admitted to the clinic’s vertigo consultation unit for the evaluation of vertigo and dizziness between January 2015 and November 2017. They had to be aged between 18 and 75 years of age, the disease onset had to have been 6 months previously or longer (defined as chronic vestibulopathy), and the participants had to have been examined by an experienced otolaryngologist with expertise in vertigo disorders. The patients’ chronic symptoms fluctuated over the day, and there were situations in which the frequency and severity of symptoms would increase, for example, when the eyes were closed, when more concentration was needed, or when the patient was stressed. No active pathology was recorded, and the patients did not complain about several single attacks of vertigo in the past combined with symptom-free intervals; there were also no signs of a central pathology nor of the acute/subacute phase of vestibular neuritis. The workup included clinical examination and a caloric labyrinth testing. The diagnoses of peripheral vestibular failure, vestibular neuritis, and vestibulocochlear failure were counted as equivalent for unilateral peripheral vestibulopathy. Exclusion criteria were the following conditions: Menière’s disease and acoustic neurinoma (to exclude conditions causing hearing problems), severely reduced hearing ability (as assessed by a dedicated hearing test), any systemic neurological, orthopedic, cardiologic, or metabolic disease, which could influence the result of the tests. Hearing tests had been performed on all of the patients—apart from some cases of mild bilateral presbyacusis, and no severe (unilateral) hearing deficits (as in Meniere’s disease) were observed. None of the participants had undergone a dedicated rehabilitation program. From the 850 patients, 81 fulfilled the named criteria and were contacted. From the contacted patients, 60 were either unwilling to take part in the study, were ineligible for MRI, or the contact data were outdated. In the end, 21 patients were invited to the test center, where earlier history and data were gathered; they were asked for their remaining symptoms and underwent a video head impulse test (vHIT) of the vestibulo-ocular reflex (VOR) and a cranial MRI. The clinical and neurootological data were then reviewed by an expert (author MD), who identified 15 patients with chronic uni- or bilateral vestibulopathy according to the international definition on unilateral and bilateral vestibulopathy [3] and presbyvestibulopathy [30] (age: 56.8 ± 10.1 years; gender: 4 females; education: 13.9 ± 1.9 years; physical activity: 1.2 ± 1.6 h/week, see Table 1). The diagnosis was based on the symptoms of chronic dizziness with gait instability in combination with the confirmatory findings in caloric testing and/or vHIT gain. Note, that caloric data were missing in one patient (P13), and vHIT data were not analyzable in two patients (P14, P15). Patient P13 had clinical symptoms of dizziness and pathological vHIT at the time of the experiment, and patients P14 and 15 had demonstrated pathological calorimetry in the acute phase and showed persisting clinical symptoms at the time of the experiment. Hence, we felt that it was safe to include these three patients in spite of their partly missing clinical data. Regarding symptom severity and HIT and caloric performance—which were not based on subjectively perceived scores—the patients were qualified as being rather mildly affected. The 15 patients were age- and gender-matched with the control participants (age: 57.6 ± 10.5; gender: 4 females; education: 16.7 ± 4.0 years; physical activity: 1.9 ± 2.4 h) in a pairwise manner, without any significant differences in any of the demographic and physical activity parameters, except for in education. Ten patients were employed when the tests were conducted, while five were either age-retired or had retired early due to the condition. Both groups received the same amount of money for their participation. The sample size was based on effect sizes and power calculations that had been obtained from our previous studies [14,25].

This study was single-blinded (analysis), cross-sectional study with one factor: group (control, vestibulopathy). All of the measurements took place in the German Center for Neurodegenerative Diseases (DZNE) from June 2018 to September 2019. An overview of the main characteristics of the patients is shown in the figure below (see Table 1). 

### 2.3. Vestibular Non-Cognitive Tasks

#### 2.3.1. Clinical Balance Test (CBT)

The CBT has also been described in detail in our previously published work [15,16,26]. Briefly, this test consists of standing on stable and unstable surfaces and walking conditions, all of which further contain sub-conditions with open and closed eyes. In total, there are 30 assessment items, and the maximal number of points that can be collected is 90, with each condition carrying a minimum of 0 and a maximum of 3 points.

#### 2.3.2. vHIT

**Figure 2:**
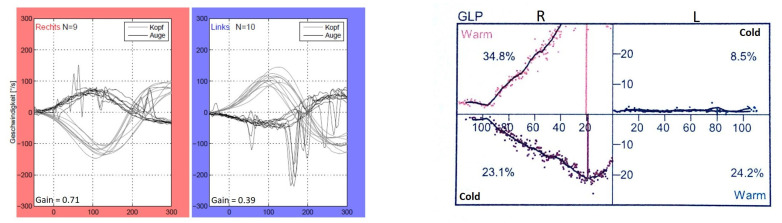
vHIT and calorimetric recordings of one patient (P11) with left unilateral vestibulopathy.

**Figure 3:**
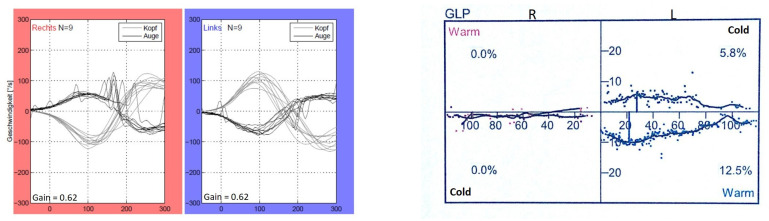
vHIT and calorimetric recordings of one patient (P9) with bilateral vestibulopathy.

According to the instructions, during the vHIT, the test participants sat in front of a wall with five manually marked points—four forming a rhombus and one in the center of the rhombus. Each participant received a pair of glasses with an eye camera to record the eye movements of the left eye. Participants were asked to focus on the central point of the rhombus, which was located in the direct field of vision. The rhombus was formed by four further points, each of which was at the same distance away from the middle point. After a calibration (looking at each point for one second several times), which served the registration of the field of view of the rhombus by the eyes, the actual test took place. The participants were instructed to fixate on the middle point of the rhombus with both eyes during the entire test. By purposeful, short, and fast head movements of approx. 15 degrees to the left and right (altogether 10 per side) according to the following sequence: “3 to the right > 3 to the left, 2 to the right > 2 to the left, 3 to the right > 3 to the left, 2 to the right > 2 to the left”, the horizontal eye movements of the test participants were recorded. The vHIT was performed using the EyeSeeCam from Interacoustics, Middelfart, Denmark. Figure 2 and Figure 3 depict representative vHIT recordings from one patient with unilateral and from one patient with bilateral vestibulopathy, respectively.

The applied normative values/reference range of the video-HIT are mainly based on studies with a large number of healthy subjects as well as with different age groups [31,32,33,34]. A VOR gain above 0.8 is classified as normal; a VOR gain below 0.7 on one side could be interpreted as a unilateral peripheral vestibular deficit; a bilateral VOR gain of between 0.8 and 0.6 in combination with an appropriate patient history and bedside examination is compatible with a so-called presbyvestibulopathy [30]; a bilaterally reduced VOR gain of below 0.6 indicates a bilateral vestibulopathy [3].

#### 2.3.3. Vestibular Caloric Stimulation

Video-controlled caloric testing was performed in the ENT clinic during the acute phase of disease. Video goggles with closed visors were put on the participants’ heads; the goggles were opened for recovery after each measurement. Participants were asked to lie in the Hallpike position (supine with the back raised about 30°). Caloric irrigation was performed with a standardized device (Variotherm plus, Atmos, Lenzkirch, Germany) in which the flow quantity was fixed and adjusted for stimulating the vestibular organ, and the stimulation duration was set to 30 s for both the irrigation with warm (44 °C) and cold (30 °C) water. As an outcome variable for the analyses, the mean of the maximal peak velocities of the slow phase of caloric-induced nystagmus for bithermal stimulation with warm and cold water was chosen. Values on each side <6°/s indicated significant vestibular hypofunction in the low frequency range [3], and values between 6°/s and 25°/s represented mild hypofunction such as in presbyvestibulopathy [30].

### 2.4. Vestibular Cognitive Tasks

#### 2.4.1. Triangle Completion Test (TCT)

The triangle completion test (TCT) from our previous studies [15,16,26] was used for the assessment of each participant’s non-visual spatial orientation abilities; for a more detailed description of the test, please refer to these studies. In short, six triangular paths were marked on the floor of a room, three going towards the left and three going towards the right, thus creating three pairs of triangular paths, with turning angles of 60°, 90°, and 120°. Each participant walked (active) and was pushed (passive) o along each of the paths one, thus resulting in 12 trials per participant in total (three to the left and three to the right multiplied by the two conditions). For the task, the participants were asked to walk back to the starting point using the shortest possible route. The main outcome variables were the distance and the angular error.

#### 2.4.2. Rotational Memory (RM)

The subject was seated in a chair that rotated (Interacoustics, Middelfart, Denmark) about an on an earth horizontal axis (left and right rotations). The participants were blindfolded, and their ears were covered. After one or more rotations in one or both directions had been executed by the software, the subject was asked to return to the initial position, by instructing the examiner on how much to rotate the chair manually until the initial position has been reached. 

The following rotations were executed twice each by the software: one, two, four, and eight rotations. After each trial, the chair was automatically rotated back to the initial position. 

### 2.5. Visual (Non-Vestibular) Cognitive Tasks

All cognitive “paper and pencil” tests were administered in a quiet testing room. There were no dropouts from the cognitive testing; therefore, all study participants took part in the tests. 

#### BIS-4—Visuo-Constructive and Spatial Abilities

Visuo-constructive and spatial abilities were assessed using two timed subtests from the test battery of the “Berlin Intelligence Structure Test” (BIS-4) [27]. The subtests measured performance within the spatial and visuo-constructive domains. 

In the first subtest, a simple drawing of a city from a birds-eye perspective was presented, where some buildings were coloured in black, and where the other buildings were coloured in white. After being allowed 90 s to memorize only the black buildings, the participants received another sheet of paper with the same city where all of the building were. Subsequently, another 90 s were allowed to recall and to mark only the black buildings from the previous sheet.

The second subtest consisted of five unfolded rectangular objects and five possible appearances for each of these objects after their sides have been folded together. Only one of the five offered solutions for each of the objects was correct. Participants were allowed 135 s to solve this subtest. Before the subtest was initiated, the participants were allowed another 70 s to solve two similar tasks and become familiar with the subtest.

We also applied another subtest of the BIS-4 to test logical-spatial thinking. In each of the eight tasks within this subset, the participants were shown three objects, with two of the objects in each set demonstrating a logical relationship, followed by a question mark where the fourth objective should have been. They were then asked to select one of five offered objects and to place it at the missing location (in place of the question mark) so that the last two objects would share the same logical connection as the first two objects

### 2.6. General Cognitive Task

#### d2-R—Attention and Concentration Abilities

In addition, to assess attention and concentration abilities, the participants also performed the revised version of the d2 test (d2-R), which has been described in detail elsewhere [28,29]. The participants were provided with a sheet of paper with the d2-R test on it and a blue ballpoint pen. The test consisted of 1 form with 798 items. Each item included the letters “d” or “p” and between one and four dashes. There were 13 different characters, 3 of which (“d” with two dashes) were target objects, with the others serving as distractors. The characters were arranged in 14 lines with 57 characters each, and lines 2–13 related to the overall result. The task was to cross out the target objects in the test. A processing time of 20 s was provided for each line, and the test was performed without a break. The researchers reminded participants to work from left to right; to start working on the next line immediately when given the instruction “next line”; and to work as quickly as possible, without making mistakes. The entire experiment, including instructions, exercises, and the task lasted for approximately15 min. The data were processed according to the Brickenkamp et al. 2010 procedure. The five parameters of the d2-R test that were extracted and that were used for analysis were as follows: (i) processed target objects (PTO), speed of work, speed during test processing (no. processed target objects); (ii) E%, accuracy during test processing (no. errors divided by no. processed target objects); (iii) CP, the number of crossed-out target objects minus the number of omission errors; (iv) EO, the number of target objects not crossed out; and (v) EC, the number of non-target objects crossed out. 

### 2.7. Structural Brain Analyses (VBM)

MR images were acquired on a 3 Tesla Siemens MAGNETOM Verio scanner (Syngo MR B17, Siemens, Munich, Germany) using a 32-channel head coil. High-resolution T1-weighted MPRAGE sequences were acquired using a 3D magnetization-prepared rapid gradient echo imaging protocol (224 sagittal slices, voxel size: 1 mm × 1 mm × 1 mm, TR: 2500 ms, TE: 3.47 ms, TI: 1100 ms, and flip angle: 7°).

Voxel-based morphometry (VBM) is a whole-brain unbiased technique for the analysis of regional GM volume and tissue changes [35]. Preprocessing involved gray-matter segmentation, template creation via DARTEL, spatial normalization to standardized Montreal Neurological Institute (MNI) space, and smoothing with a Gaussian kernel of 8 mm full width at half maximum (FWHM). Whole-brain analysis was performed first and was followed by region of interest (ROI)-analysis of the medial temporal lobe regions, including the hippocampus and the parahippocampus on both sides. 

### 2.8. Statistical Analysis

Behavioral data analysis was performed with SPSS v.21 (IBM, Armonk, NY, USA). The data were checked for assumptions of normality and homogeneity of variance. To inspect the between-group differences, an independent *t*-test or Mann–Whitney-U test (when assumptions not fulfilled) with the between-group factor group and Bonferroni or the family-wise error (FWE) correction for multiple comparisons were applied.

In the tables, the respective means with standard deviations are presented. In addition, the effect sizes (Cohen’s d) are listed. In the figures, the respective means with two standard errors of the mean are depicted. 

The data were collected and anonymized by Sabrina Sulzer. and were subsequently analyzed by Sabrina Sulzer and Milos Dordevic, who was blinded to the data collection.

## 3. Results

### 3.1. Vestibular Non-Cognitive Task (CBT) 

**Figure 4:**
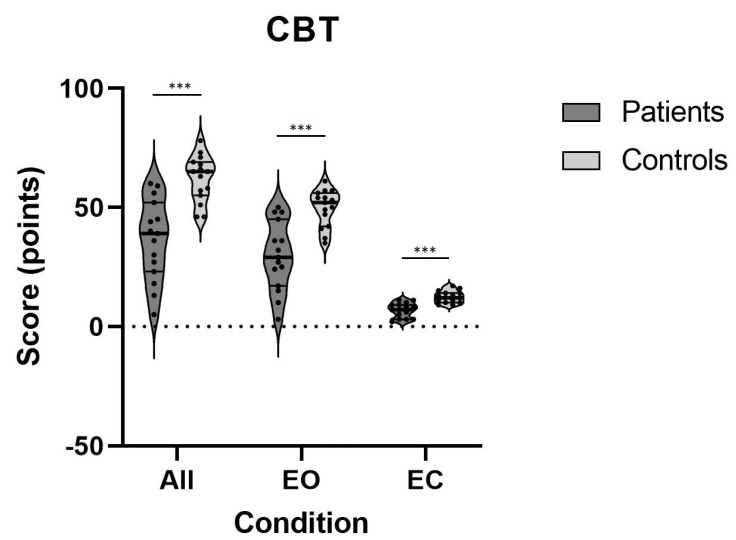
Errors on CBT on all conditions for both patients and controls; ***—*p* < 0.001; CBT—clinical balance test.

Figure 4 shows the difference between the groups of 15 patients with mild chronic vestibulopathy and their controls in the CBT for their overall scores as well as for the conditions with open and closed eyes separately. The vestibulopathy patients revealed significantly lower scores overall and on each of the conditions compared to the controls, and the effects were large (see Table 2).

### 3.2. Vestibular Cognitive Tasks (TCT and RM)

Table 3 presents data showing that patients with chronic vestibulopathy performed significantly worse on all spatial-cognitive tests that required inputs from the vestibular system compared to the control subjects, demonstrating medium to very large effect sizes.

**Figure 5:**
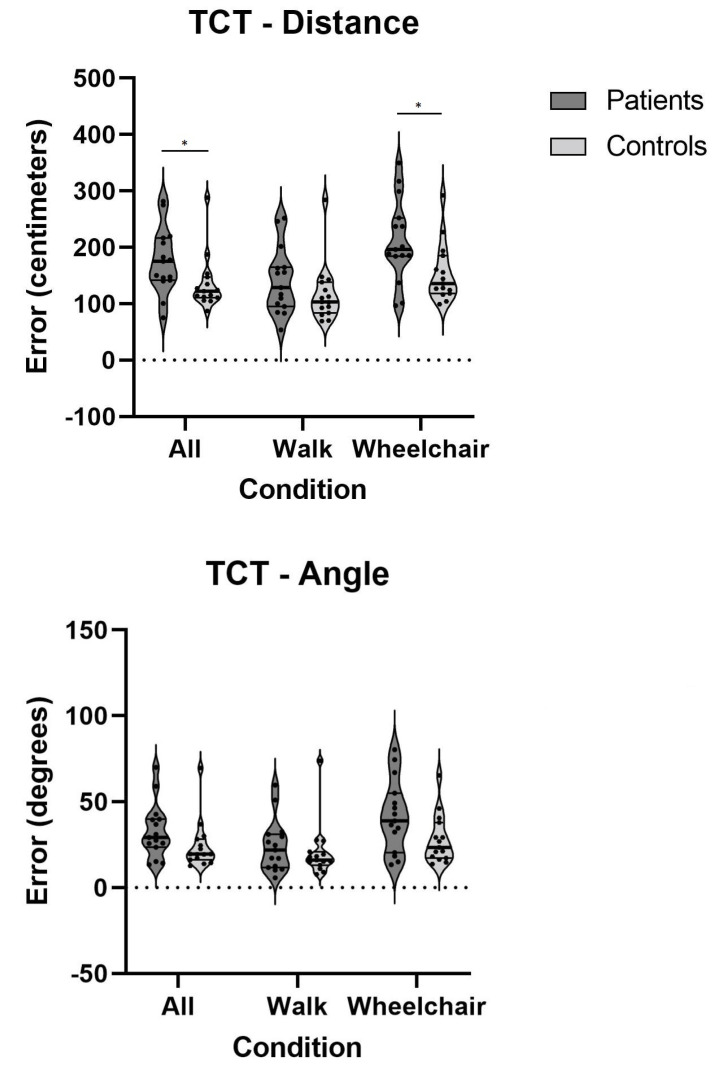
Errors on TCT on all conditions for both patients and controls; *—*p* < 0.05; TCT—triangle completion test.

In particular, the patients performed significantly worse in the wheelchair (vestibular) condition for the triangle completion test (TCT) (Figure 5), demonstrating a decreased ability to return to the starting point, which was represented by a larger distance from the point where they ended up to the original starting point. 

**Figure 6:**
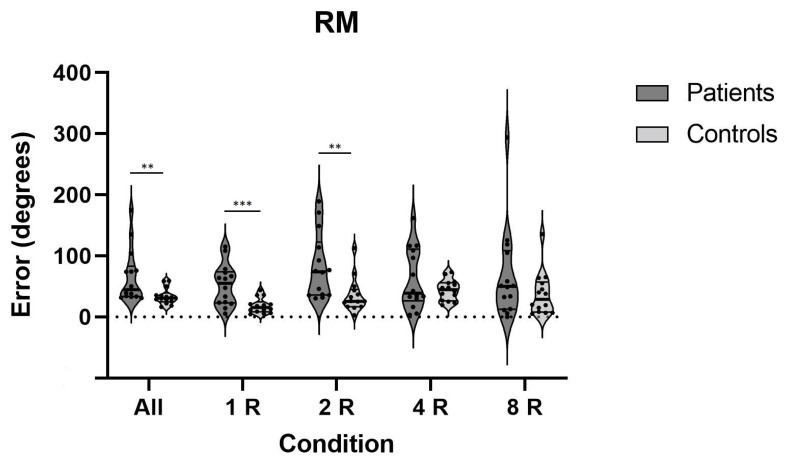
Errors on RM test on all conditions for both patients and controls; **—*p* < 0.01, ***—*p* < 0.001; RM—rotational memory.

Likewise, as presented in Figure 6, the patients’ performance on the rotational chair was overall significantly worse than it was in the controls. Their ability to detect rotational movements based on inputs from the vestibular system and to recall this movement immediately thereafter was decreased on conditions with one and two rotations. Effect sizes on the rotational memory test were medium to very large (Table 3).

### 3.3. Visual (Non-Vestibular) Cognitive Tasks (BIS-4) 

A visuo-spatial subset of the BIS-4 test was used to investigate whether the observed differences in vestibular-dependent spatial tests (TCT and RM) between the two groups could also be found on corresponding cognitive tests designed to assess visuo-spatial abilities. As revealed in Table 4, there were no significant differences in any of the visuo-spatial cognitive tasks.

### 3.4. General Cognitive Task (D2-R)

To test whether the observed differences between the two groups resulted from more general cognitive impairments (i.e., attention/concentration), we applied the D2-R test. As shown in Table 5, we found no significant differences on any of the outcomes of this attention and concentration test.

### 3.5. Whole-Brain Analysis

Using whole brain VBM analysis, we could not find any significant differences in the gray matter volumes between the vestibulopathy patients and the controls.

#### Region of Interest (ROI) Analyses

Several brain regions are known to be crucial for spatial orientation and navigation, such as the precuneus [36,37] and the medial temporal lobe [14,38]. Others are considered to be important for processing vestibular information, such as the insula [35]. For this reason, we additionally performed region of interest (ROI) analyses in these regions, and these analyses were conducted separately for each hemisphere. However, similar to the whole-brain analysis, we could not detect any significant volumetric differences between the patients and healthy controls within these regions.

## 4. Discussion

This study investigated effects of mild chronic vestibulopathy on skills and cognitive tasks that both did and did not require vestibular input. Patients with a history of proven uni- or bilateral vestibulopathy, persistent clinical symptoms, and a persisting actual deficit in the VOR were compared to healthy controls regarding their balance control ability, their performance in spatial cognitive tasks (either depended on vestibular or visual input), their general cognitive abilities, and their brain structure. As expected, the patients performed significantly worse on the tests assessing both the physical (CBT) and cognitive (TCT, RM) effects of vestibular-related functions but showed no differences compared to healthy controls in the general (D2-R) cognitive tests. With less clear predictions in mind, we found no differences in spatial visuo-cognitive (BIS-4) performance and no volumetric differences between our patients with mild uni- or bilateral vestibulopathy and the healthy controls in any of the relevant brain areas, including the hippocampus and the insula. 

Results from the clinical balance test (CBT) in the current study were within the expected direction. In our previous studies, we already discussed a link between vestibular system function and performance on the CBT [14,15,16,39]. For instance, we could show that professional ballet dancers, who intensively stimulate their vestibular system on a daily basis, scored significantly higher on the CBT compared to age- and gender-matched controls. Moreover, one month of intensive slackline-training led to improvements on this test. This is in accordance with other studies that also found that vestibulopathy patients perform worse on balancing tasks [38,40].

To test the hypothesis as to whether the processing of vestibular information in the hippocampus within the medial temporal lobe (MTL) is disturbed in chronic vestibulopathy, we ran the triangle completion and rotational memory tests. Indeed, the vestibulopathy patients performed significantly worse than the healthy controls did on both occasions. We speculated that the patients’ capacity to process vestibular inputs in the hippocampus was impaired, where both the grid and place cells are located [5,6]. The current results indicate that peripheral damage to the vestibular system causes the same deficit in non-visual spatial memory, which is also supported by the study of Xie and colleagues, who found similar effects [30]. Hence, it can be assumed that both dysfunction of the temporal lobe (as, e.g., in temporal lobe epilepsy) and vestibulopathy perturb the vestibulo–MTL axis, albeit from different ends, with similar consequences on spatial memory when visual input is blocked.

While path integration, such as in the TCT, is a well-studied function, only a few studies have assessed rotational memory up to date [7,39,41], with even less studies having bee conducted in vestibulopathy patients. Vestibular inputs play a critical role during angular rotations by converting angular motion information to distance information, a path integration process that is known to rely on the temporal lobe and its associated regions. In an earlier study, we had observed that patients with temporal lobe epilepsy performed significantly worse on this test compared to healthy controls [39]. Furthermore, in our study on participants who had undergone one month of slackline-training with closed eyes, we found an improvement on this test (unpublished data). The current study is the first to report a decrement in vestibular-related abilities on rotational memory tests in vestibulopathy patients. Nevertheless, due to a major lack of information on rotational memory abilities in humans, we are unable to make a direct comparison. 

It could have been assumed that chronic vestibulopathy may also compromise the spatial functions that rely on visual input, as vestibulopathy has been shown to entail hippocampal atrophy and because the hippocampus is considered to be the key structure for visuospatial memory. However, the existing literature on the relationship between vestibulopathy and visuo-spatial abilities remains controversial (for review see [42]). For example, in patients with chronic unilateral vestibular hypofunction and psychological distress, deficits on visuo-spatial and navigation tasks, including walking navigation, were observed [43]. Patients with bilateral chronic vestibulopathy have regularly been reported to suffer from impaired spatial cognition and even from deficits in other cognitive domains [44]. On the other hand, Hüfner and colleagues could not find any significant differences between vestibular experts and controls on a virtual Morris water maze (vMWM) task [45]. However, this could mean that a normal function cannot be improved in a way that can be seen by the vMWM. Similarly, no difference to the controls in terms of mental transformation abilities, including path reproduction, could be found one month after the patients suffering from unilateral vestibular disease had undergone surgery [46]. Moreover, no differences were reported on vMWM task performance between those suffering from left unilateral vestibular disease and the controls [13]. Keeping in mind that the right ipsilateral pathways mediate the input to the vestibular dominant, right hemisphere [47], lesions of the left labyrinth are known to cause milder deficits with better compensation [48]. Mental rotation was also reported as not being affected in unilateral vestibular disease [49]. In line with the latter studies, in the present study, we did not observe significant differences in terms of visuo-spatial tests and general cognition between our sample of chronic vestibulopathy patients, who tended to demonstrate mild deficits, and the controls. We speculate that the heterogeneity in our findings regarding visuospatial abilities in vestibulopathy patients is driven by the heterogeneity and the extent of the damage in the vestibular system across studies. Complete bilateral vestibular loss due to deafferentation is associated with stronger effects than incomplete bilateral or unilateral loss, as present in our sample of patients. 

A similar heterogeneity was seen in brain volume findings. While some studies found volumetric decrements in patients with vestibular disorders when comparing them to healthy controls [18], others could not find differences in hippocampal volumes [20] and even reported a clear dissociation between structural and functional alterations [23]. On the other side, bilateral vestibular deafferentiation in rats led to deficits on spatial memory tests without any differences in volume or neuronal number in the hippocampus. To bring additional complexity to the understanding of the underlying mechanisms, other studies found volumetric changes in regions outside of the medial temporal lobe [21,25], which was interpreted as multisensory compensation and substitution, a conclusion that was also based on the functional MRI data [50]. In our current study, we could also neither find any volumetric differences in medial temporal lobe regions when comparing the vestibulopathy patients to healthy participants nor in other regions of interest (insula, precuneus, MTL). This could speak in favor of a structure–function dissociation whereby functional deficits might take place much sooner or even without any corresponding structural brain changes. However, this is in contrast to some authors who have reported effects in patients with significant persistent vestibular deficits even after 2.5 years [25], and these findings have been attributed to the level of “damage” as the main factor for such effects. Regardless of the fact that we could not find any volumetric differences in the medial temporal lobes, it is well known that the hippocampus and its surrounding regions contain cells that are critical for processing vestibular inputs [5,8,9]. The causes for such a variability in the findings are probably methodological in nature, such as the number of participants, the type of pathology, the duration and extent of disease, the time of testing during the cause of disease, among others. A larger scale study using a more homogeneous sample of patients is necessary to shed more light on this topic.

The limitations of this study include a relatively small sample size as well as the remaining heterogeneity of the sample, which—although the deficits were generally mild—consisted of patients with different durations and extents of vestibular damage. 

In conclusion, this study provides evidence of a functional relationship between the vestibular system and brain regions that are known to process vestibular inputs, most of which are primarily located in the medial temporal lobe. The vestibulopathy patients of the current study, while showing no deficits on spatial visuo-cognitive and general cognitive assessments, scored significantly lower than the controls, not only on the clinical balance test but also on the triangle completion and the rotational memory tests, both of which were designed to assess more indirect operations and their effects on vestibular input. On the other hand, we could not find any structural differences in the regions that are known to receive vestibular inputs, such as the insula, the operculum, or the medial temporal lobe, as assessed by VBM. These findings either reflect a segregation between structure and function or a group of patients with mild vestibular hypofunction at different time points, which should be confirmed by a future large-scale study with an even more homogeneous (e.g., only unilateral damage) sample of vestibulopathy patients.5. 

## Figures and Tables

**Table 1:** Demographic and clinical characteristics of peripheral vestibulopathy patients.

Patient (P)	Age	Gender	Years of Education	Time since First Symptoms	Symptoms at the Time of Testing	Affected Side	Calorics Right/Left *	VOR Gain Right/Left
P1	58	F	13	2 years	Daily dizziness, decreased concentration	Bilateral (L > R)	6.9/1.9	0.87/0.67
P2	51	M	12	4 years	Daily dizziness	Bilateral (R > L)	2.0/5.1	0.55/0.7
P3	75	M	13	23 years	Dizziness when eyes closed	Right	1.8/6.8	0.62/1
P4	59	M	16	3 years	Daily dizziness, decreased concentration and memory	Bilateral (L > R)	8.5/3.1	0.69/0.31
P5	58	F	15	11 years	Daily dizziness, decreased concentration and memory	Bilateral (L > R)	5.0/2.1	0.86/0.67
P6	50	M	12	3.5 years	Daily dizziness, decreased concentration and memory	Bilateral	3.2/1.9	0.50/0.63
P7	66	M	14	11 years	Daily dizziness	Left	14.6/3.2	0.89/0.47
P8	57	M	15	2 years	Daily dizziness	Bilateral (L > R)	4.5/2.0	0.60/0.68
P9	52	M	16	4 years	Dizziness when stressed	Bilateral (R > L)	1.6/8.0	0.62/0.62
P10	47	F	12	5 years	Daily dizziness	Bilateral (L > R)	5.7/1.5	0.71/0.64
P11	47	M	12	11 months	Daily dizziness	Left	29.2/1.7	0.71/0.39
P12	41	M	17	9 months	Daily dizziness	Bilateral (L > R)	5.9/2.0	0.6/0.35
P13	74	M	17	2 years	Daily dizziness	Bilateral	No data available	0.62/0.62
P14	55	M	12	3 years	Daily dizziness, tendency to fall, decreased attention	Bilateral (L > R)	4.6/2.7	HIT data not analyzable
P15	75	F	13	7 years	Daily dizziness decreased concentration	Bilateral	1.1/1.5	HIT data not analyzable

* The value represents the mean of the maximal peak velocities (degree/s) of the slow phase (SPV) of caloric-induced nystagmus for stimulation with warm and cold water on each side; the sum of bithermal maximum peak SPV on each side between 6°/s and 25°/s indicates mild vestibulopathy such as in presbyvestibulopathy [31].

**Table 2:** Results of all tests and conditions of the CBT; SD—standard deviation, ***—*p* < 0.001.

Test	Condition(s)	Mean ± SD Controls	Mean ± SD Patients	*p*-Value	Effect Size (d)
Clinical Balance Test (CBT)	All Conditions	62.1 ± 9.6	36.5 ± 17.0	0.000 ***	1.86
	Open Eyes	49.7 ± 7.8	29.7 ± 14.5	0.000 ***	1.72
	Closed Eyes	12.4 ± 2.5	6.8 ± 3.0	0.000 ***	2.03

**Table 3:** Results of all tests and conditions of TCT and RM; SD—standard deviation, *—*p* < 0.05, **—*p* < 0.01.

Test	Condition(s)		Mean ± SD Controls	Mean ± SD Patients	*p*-Value	Effect Size (d)
Triangle Completion Test (TCT)	Angle	All Conditions	24.3 ± 14.2	33.0 ± 15.9	0.056	0.57
		Walk	20.6 ± 15.8	24.2 ± 15.2	0.436	0.23
		Wheelchair	28.1 ± 14.2	41.7 ± 21.0	0.067	0.76
	Distance	All Conditions	135.5 ± 48.6	175.9 ± 57.5	0.023 *	0.76
		Walk	117.0 ± 52.5	140.6 ± 58.6	0.174	0.42
		Wheelchair	154.0 ± 52.1	211.3 ± 73.1	0.019 *	0.90
Rotational Memory (RM)	All Conditions		33.8 ± 13.4	66.4 ± 44.3	0.004 **	0.99
	One Rotation		18.3 ± 12.4	52.8 ± 34.1	0.001 **	1.34
	Two Rotations		35.6 ±27.9	82.7 ± 54.1	0.002 **	1.10
	Four Rotations		43.9 ± 17.0	62.2 ± 49.6	0.769	0.49
	Eight Rotations		37.5 ± 35.4	67.9 ± 76.7	0.401	0.51

**Table 4:** Results of BIS-4 test. AN_1—Analogien (Analogies), AW_1—Abwicklungen (Unwinding).

Test	Condition(s)	Mean ± SD Controls	Mean ± SD Patients	*p*-Value	Effect Size (d)
BIS-4	Correct (in %)	47.1 ± 9.4	53.4 ± 13.2	0.187	0.55
	Wrong (quantity)	3.0 ± 1.8	2.1 ± 1.2	0.202	0.60
	Missed (in %)	52.4 ± 9.6	46.6 ± 13.2	0.202	0.50
	AN_1	18.8 ± 18.2	27.7 ± 21.5	0.367	0.45
	AW_1	24.3 ± 17.9	27.1 ± 27.9	0.902	0.12

**Table 5:** Results of D2-R test; PTO—processed target objects; E%—accuracy during test processing (no. errors divided by no. processed target objects); CP—the number of crossed-out target objects minus the number of commission/omission errors; EO—the number of target objects not crossed out; EC—the number of non-target objects crossed out.

Test	Condition(s)	Mean ± SD Controls	Mean ± SD Patients	*p*-Value	Effect Size (d)
D2-R	E%	14.3 ± 18.9	19.7 ± 15.4	0.067	0.31
	PTO	130.2 ± 33.2	122.1 ± 34.2	0.436	0.24
	CP	110.6 ± 47.6	101.2 ± 41.1	0.486	0.26
	EO	13.9 ± 15.2	17.7 ± 13.0	0.250	0.33
	EC	1.7 ± 2.9	3.5 ± 7.5	0.202	0.21

## Data Availability

All data are stored in the Otto-von-Guericke University Clinic, Magdeburg, Germany—special permission is required to access the data.
